# Hand Rehabilitation Treatment for Charcot-Marie-Tooth Disease: An Open Label Pilot Study

**DOI:** 10.4172/2155-9562.1000465

**Published:** 2018-07-30

**Authors:** Valeria Prada, S Schizzi, I Poggi, L Mori, C Gemelli, M Hamedani, S Accogli, G Maggi, M Grandis, GL Mancardi, A Schenone

**Affiliations:** 1 Department of Neurosciences, Rehabilitation, Ophthalmology, Genetics and Maternal/Child Sciences, University of Genova, Italy; 2 Ospedale Policlinico San Martino IRCCS, Dipartimento di Neurologia, Genova, Italy

**Keywords:** Rehabilitation, Charcot-marie-tooth syndrome, Rehabilitation exercise, Outcome measures, Hereditary motor, sensory neuropathies

## Abstract

**Objective:**

Charcot-Marie-Tooth neuropathy affects mainly and early the lower limbs, but hands deformities are a relevant problem, which involves the quality of life of the patients. Unfortunately, there are few studies about the evaluation of the upper limbs and very rare works about the rehabilitation. A treatment study at the moment is missing and it is important to search rehabilitation exercises to improve the dexterity and the quality of life of the patients.

**Methods:**

We recruited 9 patients with clinical and genetic diagnosis of CMT and we proposed a rehabilitation protocol which includes muscle recruitment, stretching and proprioceptive exercises for the hand with the duration of 4 weeks (two sessions for week). We evaluated the patients before and one week after the treatment with Thumb Opposition Test, Sollerman Hand Function Scale, dynamometry (tripod pinch and hand grip).

**Results:**

The rehabilitation protocol has been well tolerated and there were not dropouts. We did not observe any worsening in every scale we used. Every parameter tested showed an improvement especially in the right/dominant hand.

**Conclusion:**

This study demonstrates that this three phases treatment is well tolerated by patients, it is not detrimental for the hands status and perfectly reproducible by professionals. Moreover, this could be the basis for future randomized single blind projects.

## Introduction

Charcot-Marie-Tooth (CMT) disease is the most common inherited neuropathy with an estimated incidence of 1 in 2500, even if this number varies among different epidemiological studies [1]. CMT is caused by more than 90 genes [2] which encode for different proteins that can have a structural role in the maintenance of the myelin sheath of the peripheral nervous system, or in the axonal function or in the mitochondrial metabolism [3]. Depending on the mutation, the nerves show prominent demyelination, axonal loss or both. Patients affected by CMT complain of general weakness and distal sensory loss to the upper and lower limbs and the clinical course is usually progressive, although often slowly. All the sensory functions can be compromised, even if the proprioception is the most affected one [4].

Focusing on the hand, CMT patients along with weakness and impaired function may show two types of deformities: a) claw hand, characterized by wasted dorsal and palmar interosseous muscles and a predominance of the fingers flexors which cause a first phalanx hyperextension and a flexion of the other two phalanges; b) “simian hand”, marked by a wasting of the thenar and hypothenar muscles and a very limited opposition ability [5].

Even if patients show an important variability due to the different forms, mutations and phenotypes, hand dysfunction is a real and severe problem because it is strictly connected with the quality of life and autonomy of the patients from other people in the Activity Daily Living (ADL), as demonstrated in other diseases [6–8].

To date, rehabilitation is the only therapeutic approach to CMT [4,9,10]. Moreover, only few papers refer to the hand rehabilitation, most of them being focused to the definition of the outcome measures. Recently, a program of self-selected exercises (for the upper and lower limbs) to perform at home has shown promising results [11]. Unfortunately, standardized protocols dedicated to the hands are still lacking.

Our open label trial of hand rehabilitation with specific exercise, shows that the protocol proposed is well tolerated and effective to improve the hand functionality in patients affected by CMT and may constitute the basis for future controlled trials.

## Materials and Methods

### Patients

We recruited 9 patients (2 males and 7 females) with clinical and genetic diagnosis of different CMT types. The mean age was 54.3 ± 11.4 (range 32 to 69). None of them had other pathologies nor uncontrolled pain (Table 1). All patients were right-handed. Informed consent was obtained according to our institution policy and the declaration of Helsinki. Outcome measures were applied one day before the beginning of treatment (T0) and one week after the end of treatment (T1).

### Outcome measures (Dynamometer)

To study the variation of the strength in a objective way, we performed a maximal isometric voluntary contraction of both hands with a hand-held dynamometer (Citec CT 3001, CIT Technics BV, Groningen, The Netherlands) measuring in order tripod pinch and hand grip. Both were performed according to a standardized testing procedure [12,13]. We made three attempts, alternating the dominant and non dominant hand, with a rest of 30 seconds between the tests.

#### Thumb Opposition Test (TOT)

During the test patient touch the four long fingers with the tip of the thumb. The score range is 1-10 and is 1 for the lateral side of the second phalanx of the index finger, 2 for the lateral side of the third phalanx of the index finger, 3 for the tip of the index finger, 4 for the tip of the middle finger, 5 for the tip of the ring finger and 6 for the tip of the little finger. Then, moving the thumb proximally along the volar aspect of the little finger, the score is 7 when it touches the distal interphalangeal crease, 8 on the proximal interphalangeal crease and 9 on the proximal crease of the little finger and 10 when it reaches the distal volar crease of the hand. This test is valid only if the first stages are possible: a crawling thumb in the palm is not an opposition motion as descripted by Kapandji [14].

#### Sollerman Hand Function Test (SHFT)

The test was performed according to the author instructions [15], asking the patients to use the preferred hand.

### Rehabilitation protocol

Treatment duration was 4 weeks with a frequency of two sessions of 45 minutes per week. The duration and the frequency has been determined inspiring to some papers about the hand rehabilitation in chronic and acute diseases because, as already mentioned, literature about this argument is still lacking [16,17]. Furthermore, we choose a low frequency of session to avoid an overuse [18]. Exercises had been listed (Table 2) and three professional physiotherapists followed them scrupulously alternating each other in the sessions. Three parts composed the sessions: a first muscle recruitment phase, a second stretching phase and a last proprioceptive phase. While muscle recruitment and stretching exercises had been maintained the same over the 4 weeks, proprioceptive exercises had progressed. All exercises were performed alternating right and left hand and respecting a time of rest avoiding the overwork weakness [18]. Therapists made attention to the quality and the quantity of movements, searching the best activation of the single muscles.

### Statistical analyses

We used a paired samples *t*-test to compare the outcome measures at T0 and T1 evaluations. A *p*-value ≤ 0.05 determined significance.

## Results

All the patients followed the entire treatment and we did not have any drop out. We evaluated the results of the right and left hand.

**Figure 1:**
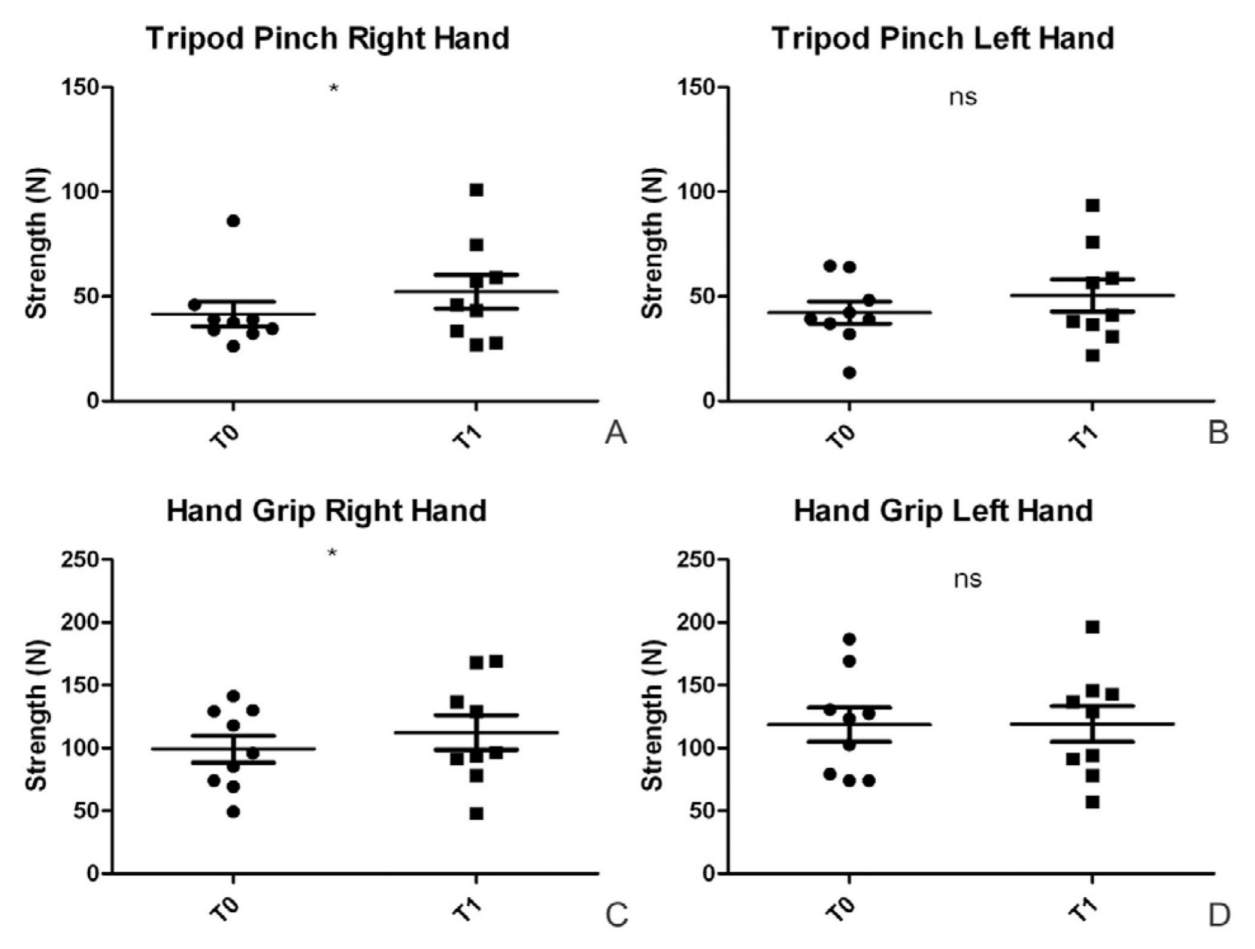
Evaluation of tripod pinch and hand grip strength before and after the treatment. A) The tripod pinch strength in the right hand shows a significant improvement (T0: 41.67±17.48 N; T1: 26±24.10 N; *p*=0.04); B) The tripod pinch strength in the left hand shows a not significant improvement (T0: 42.26±15.74 N; T1: 50.52±23.02 N; *p*=0.20); C) The hand grip strength in the right hand improves significantly (T0: 99.19±32.02 N; T1: 112.4±41.18 N; *p*=0.02); D) The hand grip strength in the left hand remains stable (T0: 118.6±40.66 N; T1: 119.3±42.74 N; *p*=0.88).

The right hand shows a significant improvement at T1 of the strength in the tripod pinch (T0: 41.67 ± 17.48 N; T1: 52.26 ± 24.10 N; *p*=0.04) and in the hand grip (T0: 99.19 ± 32.02 N; T1: 112.4 ± 41.18 N; *p*=0.02. Figure 1A and Figure 1C). The left hand also shows an improvement of the strength in the tripod pinch (T0: 42.26 ± 15.74 N; T1: 50.52 ± 23.02 N; *p*=0.20) and in the hand grip (T0: 118.6 ± 40.66 N; T1: 119.3 ± 42.74 N; *p*=0.88), although not statistically significant (Figures 1B and 1D).

**Figure 2:**
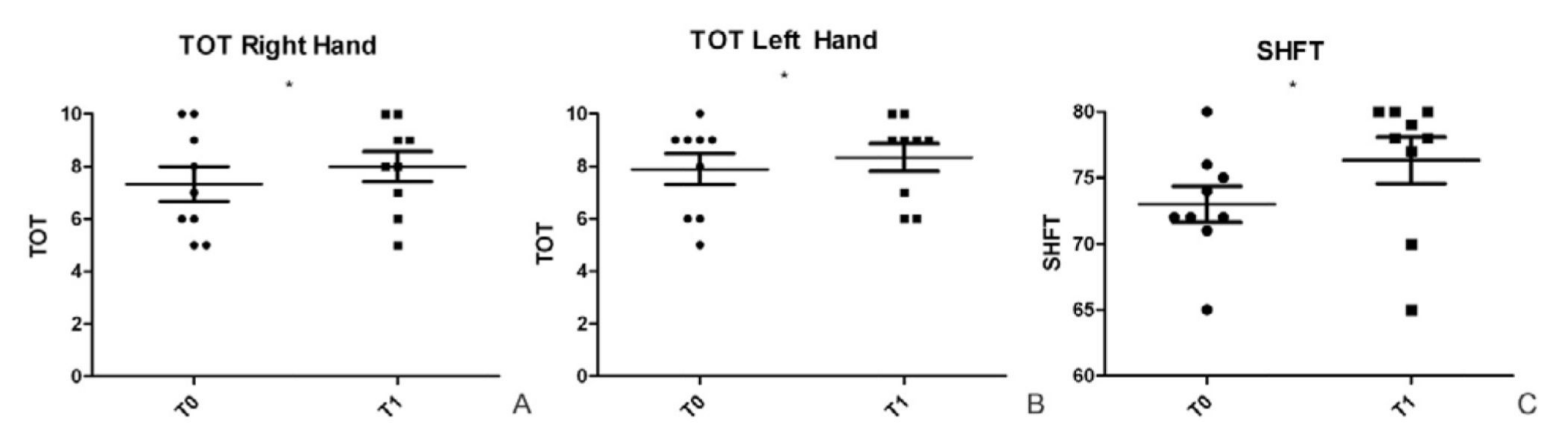
TOT and SHFT evaluation at T0 and T1. A) TOT improves significantly in right hand after the treatment (T0: 7.3±2.0; T1: 8.0±1.7; *p*=0.02); B) In the left hand TOT improves significantly after the treatment also (T0=7.8±1.76; T1=8.3±1.5; *p*=0.03); C) The SHFT, which reflects the functional impairment of the hand, shows a significant improvement after the rehabilitation intervention (T0: 73±4.1; T1: 76.3±5.3; *p*=0.02).

Interestingly, the TOT improves significantly, on average, in both hands (Right: T0: 7.3 ± 2.0; T1: 8.0 ± 1.7; *p*=0.02. Left: T0=7.8 ± 1.76; T1=8.3 ± 1.5; *p*=0.03; Figures 2A and 2B).

More importantly, the SHFT, which reflects the functional impairment of the hand, showed a significant improvement after the rehabilitation intervention (T0: 73 ± 4.1; T1: 76.3 ± 5.3; *p*=0.02) (Figure 2C).

## Discussion

Based on scattered literature data [11] and our previous personal experience, we designed a protocol for the rehabilitation of the hand in patients affected by CMT. We would aim to preliminarily demonstrate the tolerability and the efficacy of this protocol in terms of acceptance and, eventually, therapeutic capability, treating in a open label manner, 9 patients with clinical and genetic diagnosis of CMT of different types. We did not foresee the presence of placebo or of a blind study, difficult to perform in physical therapy because we were interested in quickly testing our experimental rehabilitation protocol.

The protocol, although quite demanding for the patients who had to come at the outpatients twice a week, was well tolerated and has showed a high compliance. In fact, all the subjects performed the entire program and none of them dropped out. Furthermore, all patients showed a real interest about the protocol and considered it useful and effective (we recorded the personal opinions about the protocol during the last evaluation visit on the patient folder and they were all positive). In the final interview, all of them stated to feel better after the treatment and to be satisfied. This is a very interesting result, because in our personal experience home self-administered exercises are difficult to follow and show very low compliance by the patients. Accordingly, different cases of domiciliary treatment has been reported with poor or unclear results [19,20], probably due to the severe emotional impact of the disease and the relative difficulty in respecting a program of demanding exercises [21,22]. In this view, our results underscore that a “vis-à-vis treatment” for the CMT patient is more efficient and agreeable.

The rehabilitation protocol was based on submaximal exercises, specially to avoid the overwork weakness effect [18] and at the same time improve the dexterity and the global functionality of the hand.

At the baseline, all the measured parameters show an impairment of the strength, of the articularity and the functionality of the hand compared to previously published data normal controls [18].

The rehabilitative protocol resulted in an amelioration of the performance of both hands, but the dominant hand show better and more significant results.

All the outcome measures (TOT, SHTF and dynamometer) in the right hand are significantly improved after the treatment. A slightly improvement is present also in the left hand, which is the non dominant hand for all. We can speculate that since the non dominant hand is generally less affected [18], it will take more time to recover strength and so that a longer treatment may be needed. It is also possible that the dominant hand, being more affected due to the presence of the overwork weakness [18], is easier to treat using a rehabilitation protocol based on stretching and proprioceptive exercises more than strengthening. In fact, we have strictly dosed and we paid more attention to recruit the hand muscles in a submaximal way to avoid an overwork.

The results of this open label pilot study highlight the importance of using a three components approach for the hand rehabilitation of patients affected by CMT based on recruitment, stretching and proprioception. Another key aspect is the sensitiveness of the therapist who must estimate with precision the ability of the patients and dose the applied resistance. This is obviously difficult to standardize but has to be taken into account in any rehabilitation treatment.

In summary, the results of this pilot rehabilitative trial, although open label, support that the treatment is safe, reproducible and effective in CMT patients. In fact, patients showed an high level of acceptance and compliance in completing the protocol.

## Conclusion

This is the first study addressing the use of a specific rehabilitative protocol for the upper limbs in CMT. It is sufficiently simple to be used by therapist in the physical treatment of the hand. We are aware that it is burdened by some limitations like the low number of patients, the lack of a placebo group and the significant improvement of only the non-dominant hand, thus suggesting that future randomized single blind studies are needed.

## Figures and Tables

**Table 1:** Age, sex and CMT type informations of recruited patients.

	Patients (N=9)
Mean age (SD)	54.3 (11.0)
Range of age	32-69
Males/Females	2/7
CMT1A	4 (2 Males/2 Females)
CMT1B	3 (3 Females)
CMT4C	1 (1 Female)
CMTX1	1 (1 Female)

**Table 2:** Rehabilitation protocol followed by professionals.

**Strengthening (1-4 week)**		
		Muscles involved
Abduction of the fingers with a submaximal effort	5 times per hand	Interosseous
Adduction of the fingers with a submaximal effort	5 times per hand	Interosseous
Thumb opposition with a submaximal effort	5 times per hand	Thenar eminence
Extension of the fingers with a submaximal effort	5 times per hand	Extensors
Opposition of all fingers with a submaximal effort	5 times per hand	Thenar and Hypothenar eminence
**Stretching (1-4 week)**		
Fingers flexors	5 times per finger	
Wrist flexors	5 times per wrist	
Pollicis adductor	5 times per hand	
Interosseous and lombrical (dorsal)	5 times per hand	
Interosseous and lombrical (palmar)	5 times per hand	
**Proprioception (1-2 week)**		
Turn 2 marbles in the palm per 60 sec	2 times per hand	
Theraputty manipulation: making stripes	4 times per hand	
Theraputty manipulation: little balls modeling (6 balls)	2 times per hand	
Proprioception (3-4 week)		
Turn 4 marbles in the palm per 60 sec	2 times per hand	
Theraputty manipulation: making stripes	4 times per hand	
Theraputty manipulation: little balls modeling (6 balls)	2 times per hand	
Extraction of 4 marbles from theraputty with pinch	2 times per hand	
